# Effect of Ionizing Radiation on the Microbiological Safety and Phytochemical Properties of Cooked* Malva sylvestris* L.

**DOI:** 10.1155/2018/2730713

**Published:** 2018-08-30

**Authors:** Issam Ben Salem, Souad Ouesleti, Mohamed Amine Khammassi, Abdennacer Boulila, Yassine Mabrouk

**Affiliations:** ^1^Laboratory of Biotechnology and Nuclear Technology, National Centre of Nuclear Science and Technology (CNSTN), Sidi Thabet Technopark, Ariana 2020, Tunisia; ^2^Laboratoire de Biochimie de l'Hôpital Farhat Hached, Sousse, Tunisia; ^3^Laboratory of Natural Substances, National Institute of Research and Physico-Chemical Analyses, Biotechnopole of Sidi Thabet, Ariana 2020, Tunisia

## Abstract

Nowadays, recent studies have demonstrated that plant-derived foods were characterized by their richness in bioactive phytochemicals and their consumption has a protective effect for human health. The effects of ionizing radiation on phytochemical properties of cooked* Malva sylvestris* L. (Mallow) were investigated. Irradiation increased significantly* (P<0.05)* the total polyphenols and flavonoids content of cooked Mallow. Irradiation at 2 and 4 kGy doses resulted in a significant increase in the DPPH and ABTS radical-scavenging ability of cooked Mallow extracts. There was no significant change on carbohydrate, lipid, ash, and protein content. While the mineral composition of K and Na was affected slightly after irradiation, the amounts of Mg, P, Ca, Fe, Z, and Cu remain unaffected at 2 kGy and reduced slightly at 4 kGy. The antimicrobial activity was unaffected after irradiation. Postirradiation storage studies showed that the cooked irradiated Mallow was microbiologically safe even after 20 days of storage period. Sensory properties of cooked irradiated Mallow were unaffected by the treatment. This study supports that cooking process followed by gamma irradiation did not compromise the chemical composition and sensory characteristics of Mallow.

## 1. Introduction

Aromatic and medicinal plants have been reported to contain a higher content of bioactive phytochemicals such as substantial amount of vitamins, phenolic compounds, and essential oils and thus can be used as important sources of natural antioxidants for food application and pharmaceuticals [1]. Currently, scientists are interested in developing value added products from wild and cultivated plants [2]. In fact, it is very important to increase the antioxidant intake in our nutrition, for that, there is a considerable attention to enriching food products with aromatic and medicinal plants which are considered rich in natural antioxidants [3].* Malva sylvestris* L. (Malvaceae family) known as common Mallow, is one of the most well-known medicinal herbs. Native to Europe, North Africa, and Asia, it is largely cultivated in the Mediterranean countries including Tunisia. In particular, flowers and leaves are used as a remedy for dermal infected wounds [4]. The therapeutic guide of herbal medicine in German, France, and Switzerland approved Mallow for cough [5], bronchitis, and inflammation of the mouth and pharynx [6]. Young leaves are eaten raw in salads, and leaves and shoots are consumed in soups and as boiled vegetables. Immature fruits are sucked or chewed by children, shepherds, and hunters. In Tunisia, Mallow leaves can be cooked traditionally and served as a dish. According the literature, there are many phytochemicals studies of these plants [7, 8] and to the best of our knowledge, no studies focused on the effect of ionizing radiation effect on cooked Mallow have been reported. With today's demand for high-quality convenience foods, irradiation in combination with other processes holds a promise for enhancing the safety of many minimally processed foods. Ionizing radiation has been demonstrated to be very effective for pathogen inactivation in both raw and cooked foods [9]. A 10 kGy dose is permitted by the World Health Organization for irradiation sterilization of food where toxicity testing is not necessarily involved [10]. Thus, the aims of the present report were to study the effect of ionizing radiation of cooked Mallow leaves on their microbiological safety, phytochemical, sensory, and antioxidant properties.

## 2. Materials and Methods

### 2.1. Plant Material and Sample Preparation

Samples of Mallow were collected in February 2017 from the region of Jdaida (Manouba). The collected plant material consisted of total aerial parts. The pretreatment and cooking procedures were adapted from [11]. The vegetables were washed in water and all inedible parts were removed manually. 100 g of leaves was added to 500 ml of water and cooked for 2-3 minutes at 50°C in pressure cooker thermostatically controlled. Samples were drained and rapidly cooled with cold water. Then, the samples were stored in sealed boxes (Length: 14 cm, width: 9 cm, depth: 6 cm) at 4°C. The use of only leaves is mainly due to the follow-up of the traditional cooking process of Mallow.

### 2.2. Irradiation of Cooked Mallow

The Tunisian gamma irradiation facility (at Sidi Thabet) is designed for the preservation of food stuff and sterilization of medical devices. The source consists of eight encapsulated 60Co pencils with a diameter of 9.7 mm and an overall length of 452 mm. The starting activity of the source was 99.162 kCi. The installation is equipped with a stainless steel telescopic source rack that allows obtaining a linear source of approximately 900 mm height. The source pencils are distributed circularly on a diameter of 140 mm for the upper source rack and of 80 mm for a lower one. The source rack comprises 20 housings allowing sources loading for several years. These sources are stored in dry condition in a cylindrical shield container in which they were transported. Mallow samples were exposed to gamma radiation dose of 2 and 4 kGy at a dose rate of 22.21 Gy/min and at room temperature (27±2°C). Nonirradiated (0kGy) samples were kept at 4°C and used as a control for comparative analysis. The irradiation time was 1.5 and 3 hours, respectively, for 2 and 4 kGy. Each experiment was done in triplicate.

### 2.3. Polyphenols Extraction

Irradiated Mallow cooked and control samples were dried at room temperature and ground to a fine powder. Thirteen to fourteen grams of Mallow powder from nonirradiated and irradiated samples is macerated in the presence of 140 ml of aqueous methanol solvent (80 % v/v). After filtration, the methanol solvent was evaporated at 40°C on a rotary evaporator. To prevent oxidation of the polyphenols, extraction was achieved rapidly and extracts were immediately used or conserved in darkness at -20°C until further use [12].

### 2.4. Total Polyphenols Content (TPC)

The TPC of Mallow extracts was estimated spectrometrically by the Folin–Ciocalteu method, as described by Lin and Tang [13]. Briefly, 100 *μ*L of diluted sample was added to 400 *μ*L of 1:10 diluted Folin–Ciocalteu reagent. After 5 min, 500 *μ*L of 10 % (w/v) sodium carbonate solution was added. Following 1 h of incubation at room temperature, the absorbance at 765 nm was measured in triplicate. TPC was calculated from the equation determined from linear regression after plotting known solutions of Gallic acid (10–100 ppm). Results are expressed in mg of Gallic acid equivalent (GAE) per gram of dry weight (dw) of plant material.

### 2.5. Total Flavonoids Content (TFC)

The TFC in the extracts was determined by a spectrophotometric method based on the formation of complex flavonoid-aluminium with an absorptivity maximum between 420 and 430 nm [14]. Briefly, 500 *μ*L of each extract was separately mixed with 1500 *μ*L methanol (95 %), 100 *μ*L of AlCl310 % (m/v), 100 *μ*l of sodium acetate 1M, and 2.8 mL of distilled water. The experiments were run in triplicate, and after incubation at room temperature for 30 minutes, the absorbance of the reaction mixtures was measured at 420 nm. The TFC values were determined from a standard curve prepared with quercetin (ranging from 10 to 50 *μ*g/mL final volumes) and expressed as mg quercetin equivalents (QE) / g dw.

### 2.6. Assessment of Antioxidant Capacity

#### 2.6.1. DPPH Scavenging Activity

The antioxidant activity of the polyphenolic extracts was determined using 2,2-diphenyl-1-picrylhydrazyl (DPPH) as a free radical [2]. A DPPH methanolic solution was prepared at a concentration of 4 x10-5 M. Then, 1 mL of the stock DPPH solution was added in each test tube, followed by the addition of 25 *μ*L of each polyphenolic extract. In parallel, the control was prepared containing all reagents except the polyphenolic extract and methanol was used as a blank solution. The mixture was shaken vigorously and left in the dark at room temperature. After 60 min, readings were taken using a spectrophotometer at a wavelength of 517 nm. Percent inhibition of the DPPH radical by the samples was calculated according to the formula Yen and Duh (1994):% inhibition = ((AC(o) – AS(t)) / AC(o) ∗ 100, where AC(o) is the absorbance of the control at t = 0 min and AS(t) is the absorbance of the sample at t = 60 min.

#### 2.6.2. ABTS Radical-Scavenging Assay

The radical-scavenging capacity of antioxidant for the ABTS (2,2′-azinobis-3-ethylbenzothiazoline- 6-sulphonate) radical action was determined as described by Belkhir et al. [15]. The absorbance of the reaction mixture was measured at 734 nm and compared to the antioxidant potency of Trolox used as a reference. The results were expressed in terms of Trolox. The estimate of the antiradical activity is expressed by the value of the inhibition percent (% I) calculated using the following formula: %I = [(Abs0 – Abs1)/Abs0)] x100.

### 2.7. Vitamins

The contents of individual vitamin C and vitamin E (alpha-tocopherol, beta-tocopherol, and gamma-tocopherol) were quantified by High Performance Liquid Chromatography (HPLC), based on the normalized methods EN 14130, 2003 and EN 12822, 2014, respectively [16, 17].

### 2.8. Lipids

A Soxtec System Extraction Unit Tecator was used. The crude fat was determined by extracting 0.5 g of freeze-dried sample with petroleum ether. Containers were removed and dried at 105°C, cooled, weighted, and expressed as mg/100 mg.

### 2.9. Total Proteins, Carbohydrates, Ash, and Moisture Content

Total proteins were determined as the nitrogen content by the Kjeldahl method according to the AOAC method AOAC 1995 [18]. The carbohydrate content was determined by titration in the presence of methylene blue: the Lane–Eynon method AOAC 2005 [19]. The AOAC method 942.05 was used for the determination of ash content [20]. The moisture was determined according to the AOAC 1996 [21].

### 2.10. Mineral Analysis by Atomic Absorption Spectrophotometer

Different mineral constituents (potassium [K], sodium [Na], calcium [Ca], magnesium [Mg], iron [Fe], zinc [Zn], copper [Cu], and phosphor [P]) were analyzed separately using an atomic absorption spectrophotometer.

### 2.11. Antimicrobial Effect

Whatman filter paper is used to prepare discs approximately 6 mm in diameter, which are placed in a Petri dish Mueller-Hinton solidified with agar and sterilized in a hot air oven. The loop used for delivering the antibiotics is made of 20 gauge wire and has a diameter of 2 mm. This delivers 15 *μ*L of treated and untreated cooked Mallow extract (200 mg/mL) to each disc. The bacterial concentration of* Salmonella typhimurium ATCC 14028, Escherichia coli *ATCC 8739*, Staphylococcus aureus *ATCC 6538*, Enterococcus faecium *ATCC 19434, and* Streptococcus agalactiae *ATCC 13813 is 108 CFU/mL.

### 2.12. Microbial Decontamination of Cooked Irradiated* M. sylvestris*

10 g of each sample (irradiated and nonirradiated) was homogenized in sterile Stomachers bags containing 90 ml of sterile buffered peptone water for 2 minutes using a Blender stomacher (Model 400). This mixture corresponds to the diluted 1/10 of stock suspension. A dilution series is thus prepared from 10-1 to 10-7. The fraction of surviving microorganisms in sublethally irradiated samples and unirradiated was determined according to the standards ISO 4833-1 2013 [22], for total aerobic mesophilic flora, the standard NF V 08 – 060, 2009 for fecal coliforms [23], the standard XP V 08 057 – 1, 2004 for* Staphylococcus aureus *[23], the standard NF ISO 6579, 2002 for* Salmonella *[24], and the standard ISO 7954, 2002 for molds and yeast [25].

The total mesophilic flora counting, 1 mL of each dilution, is spread on the solidified PCA medium and then incubated 72 hours at 30°C. For fecal coliforms, 1 ml of each dilution is seeded in double layer in VRBG agar medium. The inoculum is thoroughly mixed with the culture medium until solidification and incubated 24 hours at 44°C. For the counting of* Staphylococcus aureus*, 0.1 ml of the Mallow homogenate is deposited on the surface of Baird-Parker agar medium with egg yolk and potassium tellurite. Then, the inoculum is spread as fast as possible on the surface of the BP agar medium. The dishes are incubated at 37°C for 48 h.

For Salmonella enumeration, 1 ml of the preenrichment medium is transferred into 10 ml of the selenite cysteine broth medium. The medium is incubated 24 hours at 44°C.

After 24 h incubation, inoculate with a platinum loop in parallel striations on the four-side surface of the Petri dishes containing the Hektoen selective isolation medium, and the dishes are then incubated at 37°C for 24 h. The typical colonies of Salmonella are green with black centers or green or bluish colonies

For molds and yeast, the medium used for the counting of yeasts and molds is Sabouraud agar and incubation was maintained at 30°C for 3-4 days.

### 2.13. Sensory Analysis

Sensory acceptances of the cooked Mallow and irradiated cooked Mallow at 2 and 4 kGy were evaluated with 10 experimented panelists. Samples were presented in an anonymous way with a simple three-digit code. Three samples of Mallow were analyzed by panelists in individual cabins sensory evaluation. Panelists were instructed to evaluate each attribute using a ten-point hedonic scale ranging from “dislike extremely” to “like extremely”. Six different parameters were used to grade the overall quality in terms of color intensity, herbaceous smelling, flavor, melting texture, cooking taste, and overall acceptance. The proposed question was: How much do you like this product on a scale of 1 to 10, where 1 = dislike extremely, and 10 = like extremely?

### 2.14. Statistical Analysis

The results of this work were analyzed using SPSS software, version 20, by an analysis of variance test (ANOVA) to compare different means between the control samples (nonirradiated) and irradiated at 2 and 4 kGy.

## 3. Results and Discussion

### 3.1. Effect of Ionizing Radiation on TPC and TFC

It is well known that phenolic and flavonoids substances contribute directly to the antioxidant activity of plant materials. In fact, phenolic compounds exhibit considerable free radical-scavenging activities (through their reactivity as hydrogen-donating or electron-donating agents) and metal ion-chelating properties. The herein obtained TPC and TFC of the unirradiated cooked Mallow were, respectively, 186.59±14,55 mg GAE/g dw and 12,17 ± 3,88 (mg QE/g dw) (Table 1). The obtained results are in agreement with those reported by [7, 25]. The TPC of the extracts obtained from irradiated cooked Mallow at 2 kGy increases significantly (*P*<0.05) by 13.8% (212.4 ± 11.7g /g dw) compared to the unirradiated ones (Table 1). This increase is more pronounced for the samples treated at 4 kGy reaching the rate of 104 % (382.25 ± 19.35 /g dw). The same trend was observed for TFC and a pronounced increase, reaching 5 and 7 times, respectively, for 2 and 4 kGy in irradiated cooked Mallow compared to the unirradiated samples suggesting that flavonoids are less radioresistant than the other phenolic classes. Harrison and Were [26] studied the irradiation effects of almond skin extracts and found that TPC increased at 4 and 12.7 kGy. A similar increase of TPC and TFC was reported in the literature for irradiated Purslane (Portulaca oleracea) Plant [27] and Dill herb irradiated at 2, 4, and 8 kGy [28]. This increase of the TPC is due to the release of phenolic compounds from the glycosidic components and degradation of the larger phenolic molecules into smaller ones by gamma irradiation [26]. In addition, this increase is probably related to the effect of the irradiation, which breaks down the polyphenol chemical bonds and consequently induces the release of low molecular weight and soluble phenols. Similar observations have been reported for different plant material treated with different doses of ionizing radiation [29].

### 3.2. Effect of Ionizing Radiation on the Antioxidant Capacity

Table 1 showed a significant increase* (P<0.05)* of the antioxidant activity value in the irradiated cooked Mallow at 2 and 4 kGy. The EC50 calculated from the calibration curve DPPH = f (Trolox) of irradiated cooked Mallow at 2 and 4 kGy were, respectively, 150.63 ± 0.65*μ*g/mL and 101.79 ± 0.17*μ*g/mL, significantly lower* (P<0.05)* than that of unirradiated sample (158.81 ± 3,62*μ*g/mL) (Table 1). The same trend was observed in the ABTS radical-scavenging activity. Indeed, the EC50 calculated from the calibration curve ABTS= f (Trolox) were 61.1± 0.9*μ*g/mL and 54.81 ± 0.28*μ*g/mL, respectively, for 2 and 4 kGy (Table 1), significantly lower* (P<0.05)* than that of unirradiated sample (65.96±0.56*μ*g/mL). The significant increase in TPC was thus suggestive of their enhanced antioxidant properties. Similar report funded by Mohammad Akbari et al. [30] showed that ionizing radiation leads to an increase of TPC followed by an increase of antioxidant property in three different Persian pistachio nuts.

### 3.3. Effect of Ionizing Radiation on Vitamin C, E, Carbohydrates, Lipids, and Proteins

The nutrient composition is depicted in Table 1. The vitamin C content (24.32 mg/100 g) was higher than those reported by** Barros et al. **[7]. The carbohydrates, lipids, proteins, and ash content were similar to those described by the same authors. Therefore, the cooked Mallow is an excellent source of antioxidant phenols and flavonoids, being vitamin E (*α*-tocopherol) the most abundant component.

Studies on the effects of ionizing radiation in macronutrients revealed no significant difference in total carbohydrates and lipids between irradiated (2 and 4 kGy) and nonirradiated samples (Table 1). Data obtained by other authors also showed that gamma irradiation, using a dose up to 10 kGy, did not induce significant loss on lipid and carbohydrates content [10, 31]. The ash and moisture content were stable after irradiation. Significant increase in quantity of proteins was observed after irradiation at 2 and 4 KGy (Table 1). This increase could be attributed to the fact that the gamma irradiation can lead to the degradation or polymerization of protein enhancing the solubility of nitrogen [32]. Vitamins E and C appeared to be stable after irradiation since no loss was observed in an irradiated cooked Mallow.

### 3.4. Effect of Ionizing Radiation on Mineral Composition

Mineral element's contents of the irradiated and unirradiated cooked Mallow are shown in Table 2. The elements K, P, Na, Mg, and Ca were the major inorganic constituents, while Fe, Zn, and Cu were also present as minor constituents. The values reported for these elements in this study were in agreement with the findings of Hiçsönmez et al. [33]. The Na and K concentration in the control were, respectively, 460.558±20.149 and 952.934±36.413 mg/ 100 g which was significantly reduced at 2 and 4 kGy (Table 2).

While the finding reported by Sanni et al. [3] indicating that Na and K in irradiated Sorrel Seeds were dramatically reduced after 2.5 and 5 kGy, our results showed a slight decrease in these elements and the negative effect of irradiation on sodium and potassium may not be sufficient reason to foreclose the use of ionizing radiation on cooked Mallow. However, the concentrations of Mg, P, Ca, Fe, Z, and Cu were not affected at 2kGy and reduced slightly at 4 kGy (Table 2).

### 3.5. Microbial Decontamination

Table 3 shows microbial counts measured by plate method in control and irradiated cooked Mallow. The initial mean populations of the total aerobic mesophilic flora and total coliforms were 3×102 CFU/g and 103 CFU/g, respectively (Table 3). These concentrations are low due to the cooking process used before irradiation. The pathogenic* Salmonella*,* Staphylococcus aureus *bacteria, and fecal coliforms were absent in cooked Mallow samples. Samples that were irradiated at 2 and 4 kGy did not show any molds and yeast count after 20 storage days (Table 3). This result was in agreement with findings reported by Farkas indicating that molds, fungi, and coliforms are eliminated by doses lower than those required for bacteria [9]. Previous studies indicated the minimum dose as low as 4-5 kGy will destroy these organisms. The ionizing radiation at 2 and 4 kGy, compared to the control sample, reduced considerably the amount of total mesophilic bacteria and total coliforms in 10 and 20 days of storage to the permissible level recommended by the World Health Organization [9]. Thus, the cooking process followed by ionizing radiation at low doses 2 and 4 kGy improves the microbiological safety of cooked irradiated Mallow.

### 3.6. Effect of Ionizing Radiation on Antimicrobial Activity

The analysis of cooked Mallow antimicrobial activity was investigated immediately after irradiation at 2 and 4 kGy. The mean of zone inhibiting growth (ZIG) for irradiated cooked Mallow extract was particularly unchanged (*P*<0.05) at 2 and 4 kGy (Table 4) and confirms that the irradiation dose of 2 and 4 kGy has no significant effect on the antimicrobial activities of an irradiated cooked sample against gram positive bacteria, i.e.,* Staphylococcus aureus, Enterococcus faecium, *and* Streptococcus agalactiae *ATCC, and gram negative bacteria like* Salmonella typhimurium *and* Escherichia coli*. Previous works in concordance with our results demonstrated that pharmacological activity of medicinal herbs has been found satisfactory after microbiological decontamination by irradiation [34]. In addition, pharmacological tests of Brazil medicinal herbs concluded identical therapeutic action as unirradiated preparations after exposure to a dose of 10, 20, and 30 kGy of ionizing radiation [35]. Thus, the antimicrobial activity of bio-actives substances in Mallow did not change significantly after cooking process followed by ionizing radiation.

### 3.7. Effect of Ionizing Radiation on Sensory Characteristics

**Figure 1:**
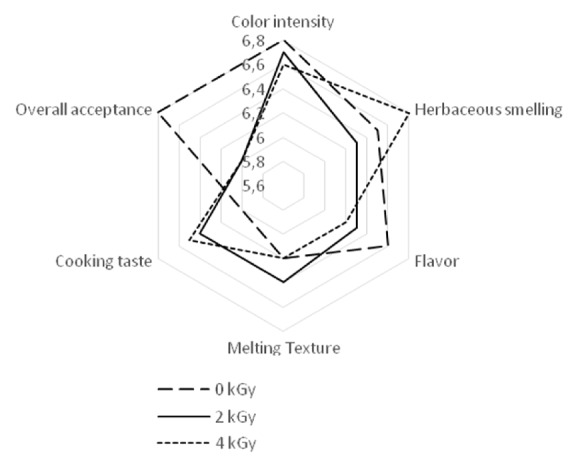
Mean sensorial ratings of untreated (0kGy) and cooked irradiated Mallow at 2 and 4 kGy.

Testers were invited to express a judgment of pleasantness ranging from “1 Dislike Very Much” to “10 like Very Much” to indicate their preferences. Mean sensorial ratings for all samples irradiated at 2 and 4 kGy received good overall acceptance scores, not significantly* (P<0.05)* different from unirradiated samples. The lowest mean score was 6 on a scale of 1 to 10 (Figure 1). Given these results, it can be asserted that irradiation at doses 2 and 4 kGy might well apply for decontamination of cooked Mallow without adversely affecting their sensory attributes.

## 4. Conclusions

In this work, the effect of cooking process followed by ionizing radiation at low doses of* M. sylvestris* was investigated. As summarized in supplementary materials (available here) the results obtained from this study confirm the significant increase of TPC and TFC after ionizing radiation. Antioxidant response was also manifested in the increase of DPPH and ABTS scavenger ability at 2 and 4 kGy applied doses. The antimicrobial activity of irradiated cooked Mallow was significantly unaffected at 2 and 4 kGy. The mineral composition was slightly affected in K and Na amount after irradiation; however, the amount of Mg, P, Ca, Fe, Z, and Cu was unaffected at 2kGy and reduced slightly at 4 kGy. The nutraceutical properties of irradiated cooked Mallow were not affected after irradiation. Therefore, cooking process followed by ionizing radiation improves the microbiological safety and maintaining sensory characteristics or even enhancing the antioxidant activity. It may emerge as one of the important techniques for preserving or improving the nutritional effect of the edible medicinal plant.

## Figures and Tables

**Table 1:** Polyphenols and flavonoids content of untreated and irradiated Mallow at 2 and 4 kGy (n=3).

**Doses (kGy)**	**0**	**2**	**4**
**TPC (mg EAG/g dw )**	186.59^**a**^ ± 14.55	212.4^**b**^ ± 11.7	382.25^**c**^± 19.35

**TFC (mg QE/g dw)**	12.17^**a**^ ± 3.88	65.80^**b**^ ±16.75	92.53^**c**^± 10.45

**DDPH EC**_**50**_ ** (** ***μ*** **g/ml)**	158.81^a^±3.62	150.63^b^ ±066	101.8^c^±0.2

**ABTS EC**_**50**_ ** (** ***μ*** **g/ml)**	65.96^a^±0.56	61.1^b^±0.9	54.81^c^±0.43

**Vitamin C (mg/100 g)**	24.32^a^±0.67	25.53^b^±0.89	26.67^c^±0.02

**α** **-tocopherol (mg/100 g)**	87.94^a^±1.32	86.54^a^±1.02	87.53^a^±0.54

**β** **-tocopherol (mg/100 g)**	5.56^a^±0.86	5.34^a^±0.81	5.32^a^±0.84

**γ** **-tocopherol (mg/100 g)**	15.54^a^±0.32	14.89^a^±1.25	14.68^a^±1.53

**Carbohydrates (g/100g)**	80.65^a^±0.37	81.05^a^±0.57	79.76^a^±0.83

**Lipids (g/100g)**	2.34^a^±0.03	2.28^a^±0.12	2.31^a^±0.04

**Proteins (g/100g)**	14.21^a^±1.01	15.5^b^±1.5	16.4^c^±1.42

**ash (g/100g)**	12.98^a^±0.02	12.56^a^±0.76	12.78^a^±0.83

**Moisture (**%**)**	75.76^a^±0.53	75.21^a^±0.32	75.01^a^±0.51

Values followed by the same letter along the row are not significantly different (P<0.05).

**Table 2:** Mineral composition of untreated and irradiated cooked Mallow at 2 and 4 kGy on dry weight basis mg/100 g (n=3).

Dose (kGy)			

Minerals	0	2	4

Potassium	952.934^**a**^±36.413	749.153^**b**^±27.085	631.293^**c**^±4.328

Sodium	460.558^**a**^±20.149	336.712^**b**^±36.828	235.782^**c**^±10.733

Phosphor	379.967^**a**^±7.611	369.832^**a**^±9.545	330.963^**b**^±3.155

Copper	2.416^**a**^± 0.198	2.425^**a**^±0.076	1.955^**b**^±0.091

Zinc	6.646^**a**^± 0.078	6.564^**a**^±1.234	5.014^**b**^ ±0.029

Magnesium	189.160^**a**^±3.925	188.712^**a**^±1.560	145.419^**b**^±2.592

Iron	16.137^**a**^±0.639	16.384^**a**^ ±0.735	12.791^**b**^±0.997

Calcium	199.889^**a**^±2.541	197.97^**a**^±1.93	161.138^**b**^±0.637

Values followed by the same letter along the row are not significantly different (P<0.05).

**Table 3:** Effect of postirradiation on microbial load (CFU/g) of cooked Mallow during 20 days of storage at 3°C ± 1°C (n=3).

**Dose (kGy)**	**Total aerobic mesophilic flora (CFU/g)**			**Fecal coliforms (CFU/g)**			**Molds and yeast (CFU/g)**			***Staphylococcus aureus *(CFU/g)**			***Salmonella *(CFU/g)**			**Total coliforms (CFU/g)**		
Days	0	10	20	0	10	20	0	10	20	0	10	20	0	10	20	0	10	20

0	3×10^2^±30	4×10^3^±52	2×10^5^±34	Abs*∗*	Abs	Abs	1	4	36 ± 5	Abs	Abs	Abs	Abs	Abs	Abs	10^3^±22	4×10^3^±28	4×10^4^ ± 62

2	<10	19	2×10^2^±38	Abs	Abs	Abs	0	0	0	Abs	Abs	Abs	Abs	Abs	Abs	<10	<10	10^2^± 51

4	<10	13	10^2^ ±25	Abs	Abs	Abs	0	0	0	Abs	Abs	Abs	Abs	Abs	Abs	<10	<10	10

**Table 4:** Antimicrobial activities of untreated and cooked irradiated Mallow under 2 and 4 kGy dose. The zone inhibition growth (ZIG) was measured in triplicate (n=3), SD= standard deviation.

	**Irradiation dose (kGy)**		

**Strains**	**0**	**2**	**4**

	**ZIG (mm)**		

***Staphylococcus aureus ATCC 6538 G(+)***	7±2^a^	8±3^a^	8±1^a^

***Salmonella typhimurium ATCC 14028 G(-)***	11.5±1^a^	10±2^a^	10±3^a^

***Enterococcus faecium ATCC 19434 G(+)***	12±2.5^a^	11±3^a^	9.5±4^a^

***Streptococus B G(+)*ATCC 13813**	7±2^a^	8±1.5^a^	7±2^a^

***Escherichia coli G(-) ATCC *8739**	**7**±0.5^a^	**7**±1^a^	**7**±2^a^

Values followed by the same letter along the row are not significantly different (P<0.05).

## Data Availability

The data used to support the findings of this study are available from the corresponding author upon request.

## Abbreviations

KGy: KilloGray
DPPH: 2,2-diphenyl-1-picrylhydrazyl
ABTS: 2,20-azino-bis(3-ethylbenzothiazoline-6-sulfonic acid)
Trolox: 6-hydroxy-2,5,7,8-tetramethyl chroman-2-carboxylic acid
TPC: Total polyphenols content
TFC: Total flavonoids content
GAE: Gallic acid equivalent
QE: Quercetin equivalent
HPLC: High Performance Liquid Chromatography
AOAC: Official Method of Analysis Chemistry
CFU: Colony Forming Unit.

## Supplementary Materials

Supplementary Materials Graphic summary description. The cooked Mallow stored in sealed boxes was irradiated at 2 and 4 kGy in Tunisian 60Co gamma irradiation facility. The starting activity of the source was 99.162 kCi. The results obtained from this study confirm the significant increase of TPC after ionizing radiation at 2 and 4 kGy. Global acceptance ratings for all samples irradiated at 2 and 4 kGy received good overall acceptance scores, not significantly different from unirradiated samples.
