# Supplementary figures and images for: Effect of Ionizing Radiation on the Microbiological Safety and Phytochemical Properties of Cooked Malva sylvestris L
Source: Biomed Res Int. 2018 Aug 30;2018:2730713. doi: 10.1155/2018/2730713 (PMC6136492; doi:10.1155/2018/2730713)

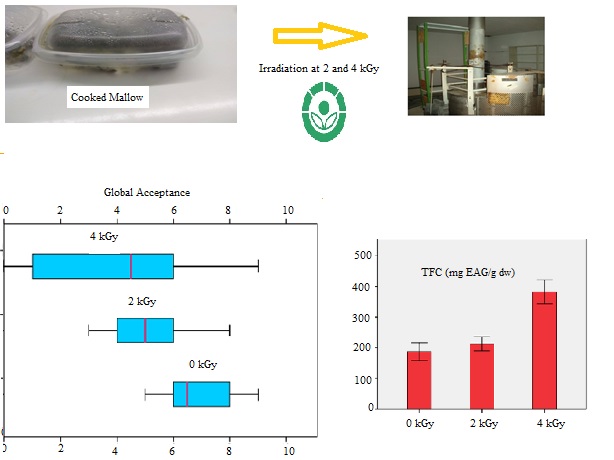

Supplement: Supplementary Materials — Graphic summary description. The cooked Mallow stored in sealed boxes was irradiated at 2 and 4 kGy in Tunisian 60Co gamma irradiation facility. The starting activity of the source was 99.162 kCi. The results obtained from this study confirm the significant increase of TPC after ionizing radiation at 2 and 4 kGy. Global acceptance ratings for all samples irradiated at 2 and 4 kGy received good overall acceptance scores, not significantly different from unirradiated samples. [file 2730713.f1.jpg]
